# Supplementary material for: Telomere Maintenance Pathways in Lower-Grade Gliomas: Insights from Genetic Subtypes and Telomere Length Dynamics
Source: Int J Mol Sci. 2025 Apr 28;26(9):4175. doi: 10.3390/ijms26094175 (PMC12071676; doi:10.3390/ijms26094175)
Supplement: Supplementary file 1 [file ijms-26-04175-s001.zip › ijms-3561274-supplementary.pdf]

Telomere Maintenance Pathways in Lower-Grade Gliomas: Insights from Genetic Subtypes and Telomere Length Dynamics

Meline Hakobyan[1],Hans Binder[2,3] and Arsen Arakelyan[1*]
1 - Bioinformatics Group, Institute of Molecular Biology NAS RA, Yerevan, Armenia
2 - Interdisciplinary Centre for Bioinformatics, University of Leipzig, Leipzig, Germany
3 - Armenian Bioinformatics Institute, Yerevan, Armenia

*Corresponding author: Arsen Arakelyan, E-mail: aarakelyan@sci

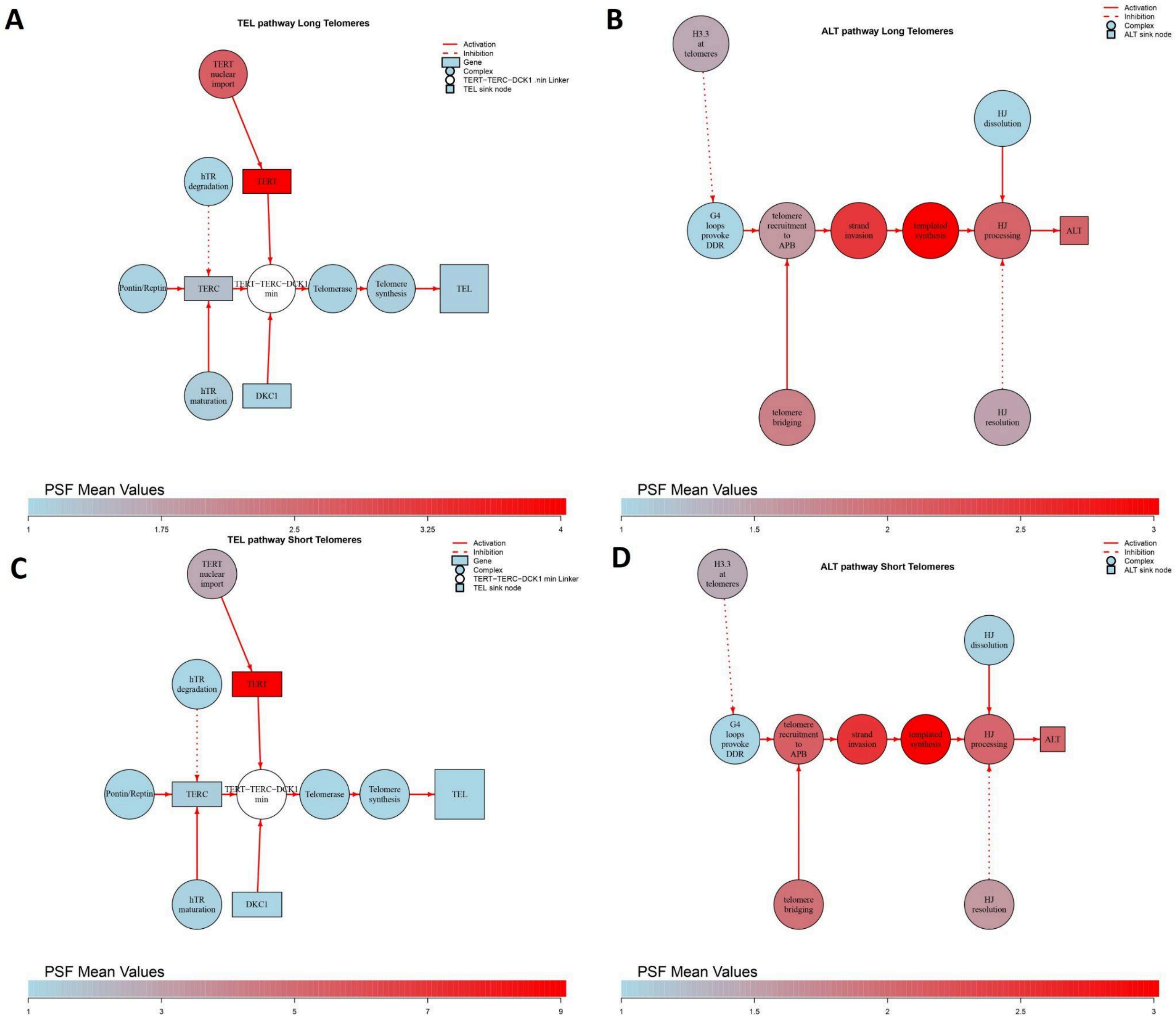

Figure. S1 Comparison of TEL and ALT pathway activity between Long and Short telomere states.

A, C the TEL pathway activity in cells with long **(A)** and short **(C)** telomeres. **B, D** ALT pathway

activity in cells with long **(B)** and short **(D)** telomeres. Solid arrows indicate activation, while dotted arrows represent inhibition. The size and shape of the nodes correspond to specific components: genes, TEL sink nodes, and complexes (e.g., TERT–TERC–DKC1 mini linker). The PSF mean values, represented by the color gradient (blue to red), indicate low to high pathway activity.

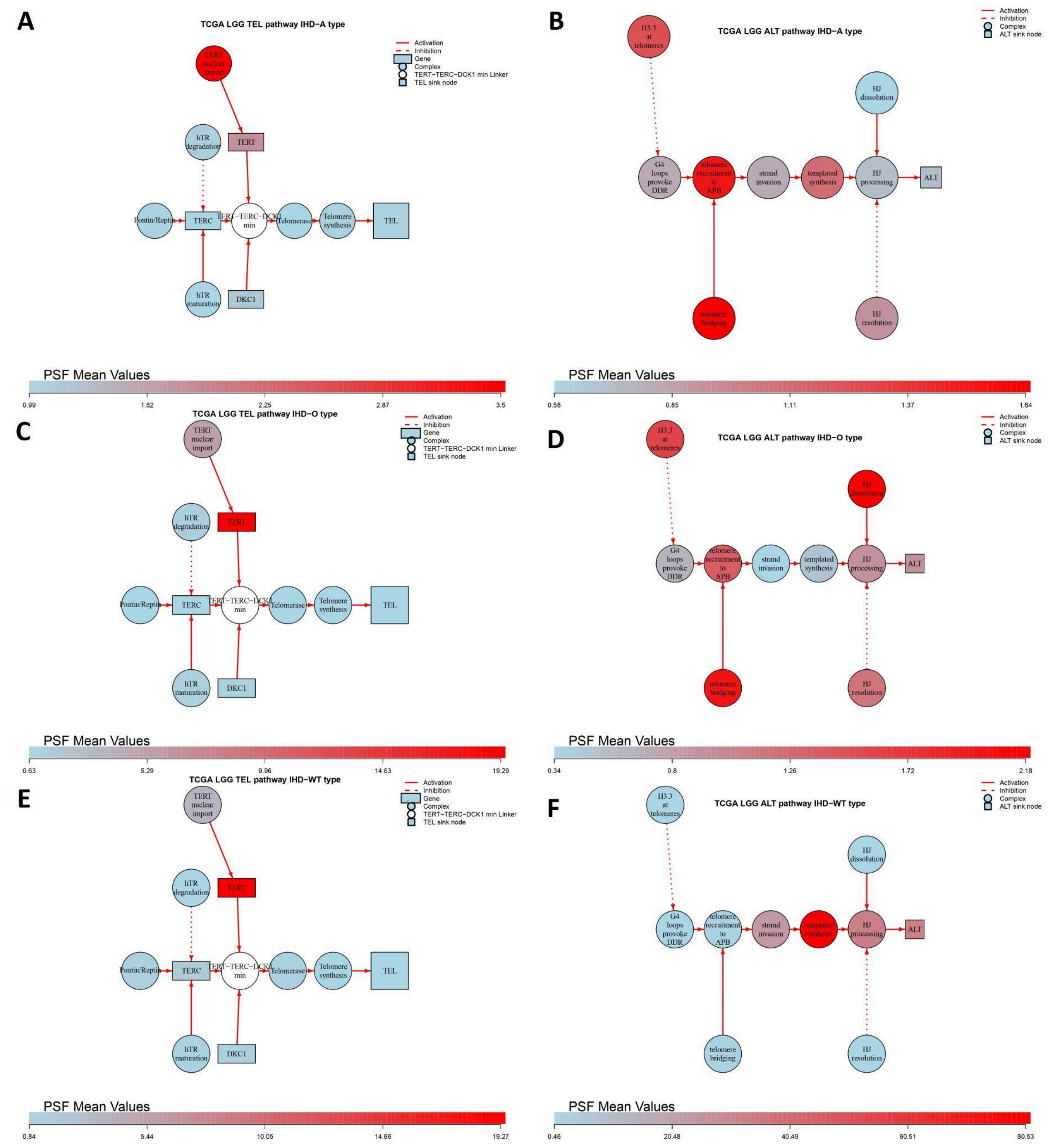

Figure. S2 Comparative Analysis of the TCGA LGG TEL and ALT Pathway Activity Across CGGA LGG IDH Subtypes.

A, C, E Visualization of the TEL pathway activity states for CGGA IDH-A, IDH-O, and IDH-WT subtypes, respectively. Pathway topology highlights key nodes involved in telomere elongation, including the activation of TERT and TERT nuclear importer. Node color intensity represents PSF mean values, with red indicating higher activity and blue indicating lower activity.

B, D, F Visualization of the ALT pathway activity states for CGGA IDH-A, IDH-O, and IDH-WT subtypes, respectively. The ALT pathway topology emphasizes key components, such as telomere recruitment to APB and strand invasion. Dotted arrows show inhibition, while solid arrows indicate activation. Node color and gradient intensity correspond to PSF mean values, showcasing distinct pathway activity patterns among subtypes.

Color gradients in the PSF scale bar range from low (blue) to high (red) PSF mean values, providing quantitative insight into the differential pathway activity.

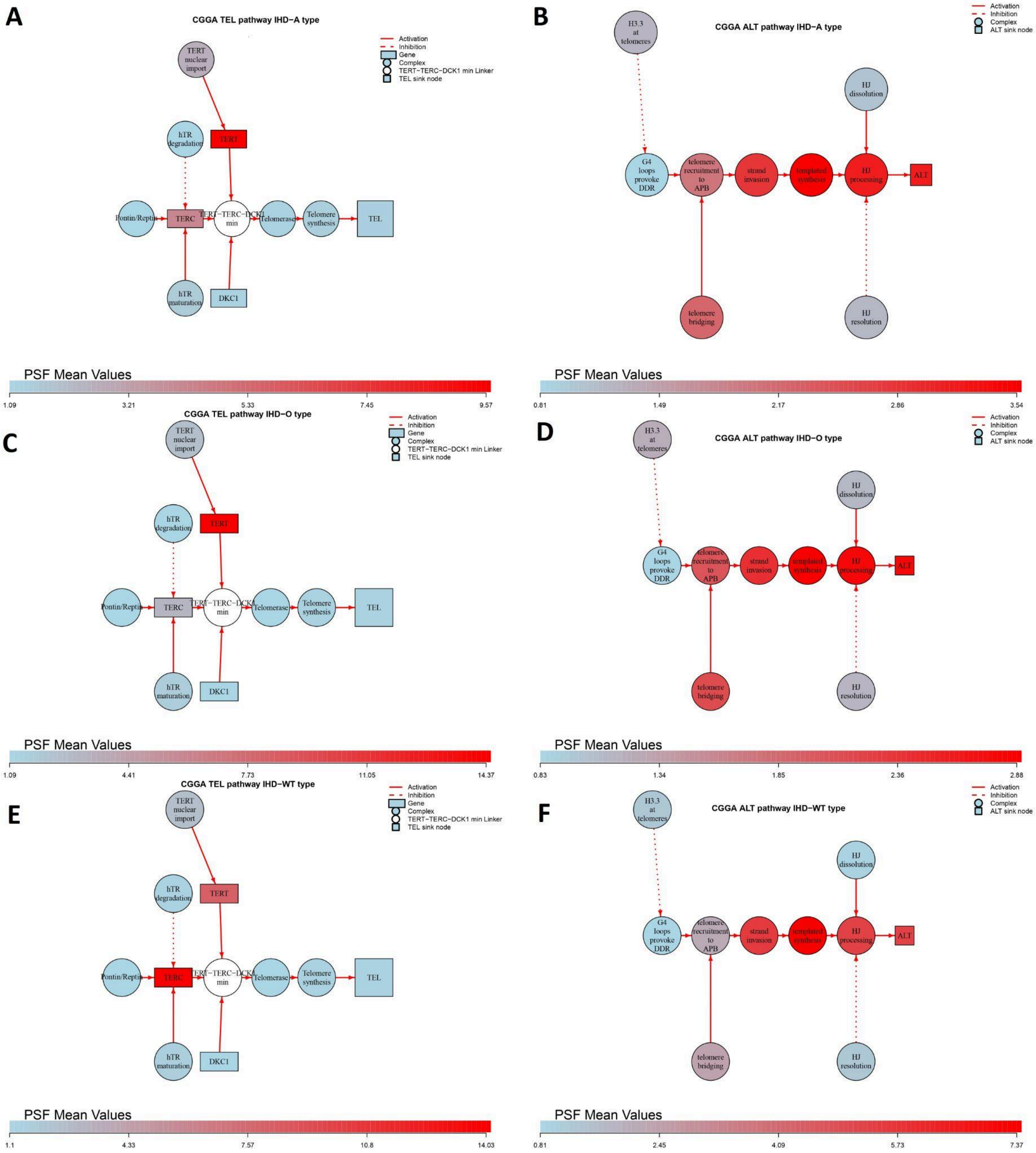

Figure. S3 Comparative Analysis of TEL and ALT Pathway Activity Across CGGA LGG IDH Subtypes.

A, C, E Visualization of the TEL pathway activity states for CGGA IDH-A, IDH-O, and IDH-WT subtypes, respectively. Pathway topology highlights key nodes involved in telomere elongation, including the activation of TERC and TERT. Node color intensity represents PSF mean values, with red indicating higher activity and blue indicating lower activity.

B, D, F Visualization of the ALT pathway activity states for CGGA IDH-A, IDH-O, and IDH-WT subtypes, respectively. The ALT pathway topology emphasizes key components, such as HJ processing and strand invasion. Dotted arrows show inhibition, while solid arrows indicate activation. Node color and gradient intensity correspond to PSF mean values, showcasing distinct pathway activity patterns among subtypes.

Color gradients in the PSF scale bar range from low (blue) to high (red) PSF mean values, providing quantitative insight into the differential pathway activity.

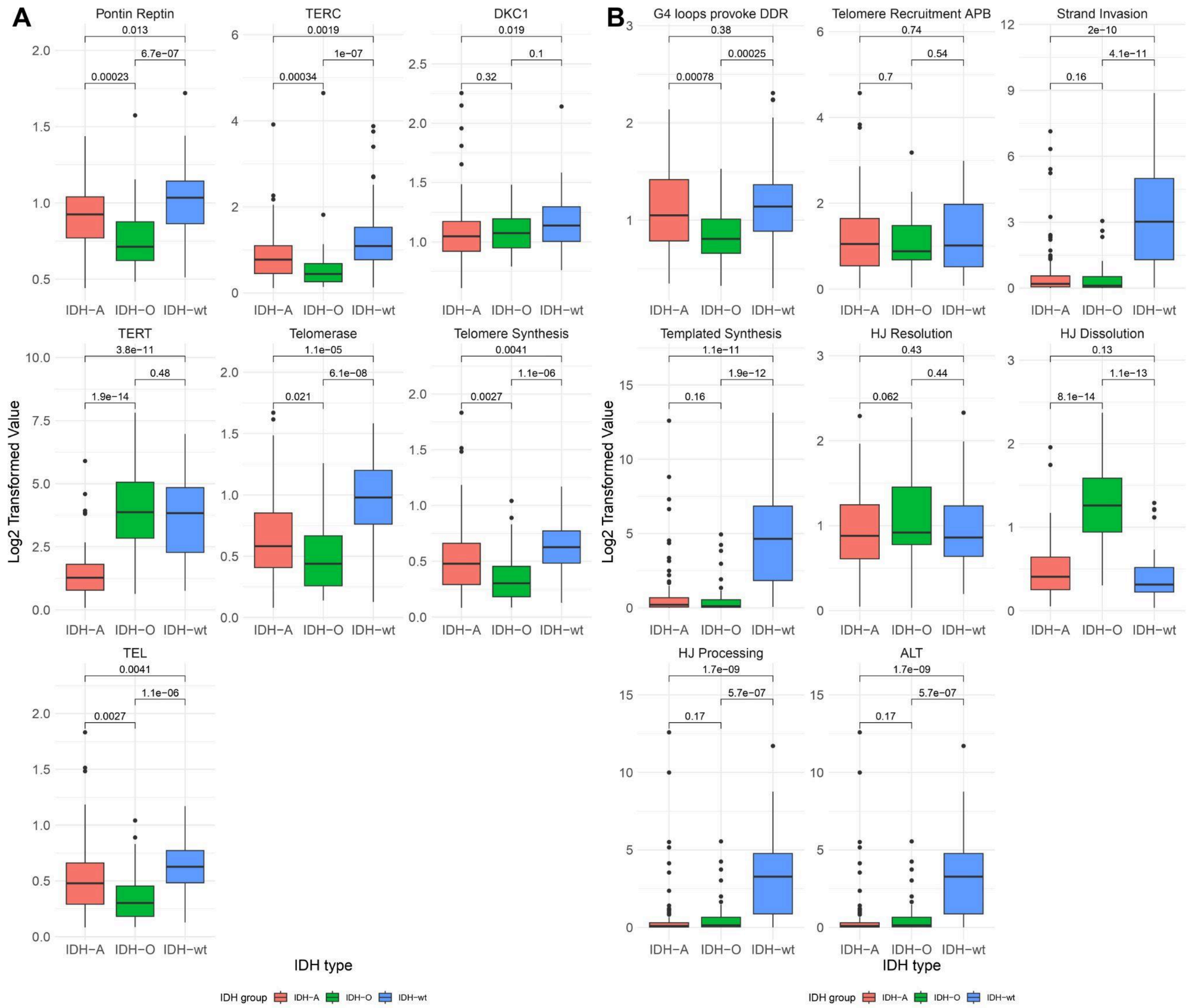

Figure. S4 The LGG TEL and ALT pathway branch activity across IDH subtypes.

(A) The TEL and **(B)** The ALT pathway Log2-transformed PSF values of key branches across the IDH subtypes (IDH-A, IDH-O, IDH-wt). Statistical comparisons were performed between telomere length groups within each IDH subtype. Statistical significance was assessed using the Mann-Whitney U test.

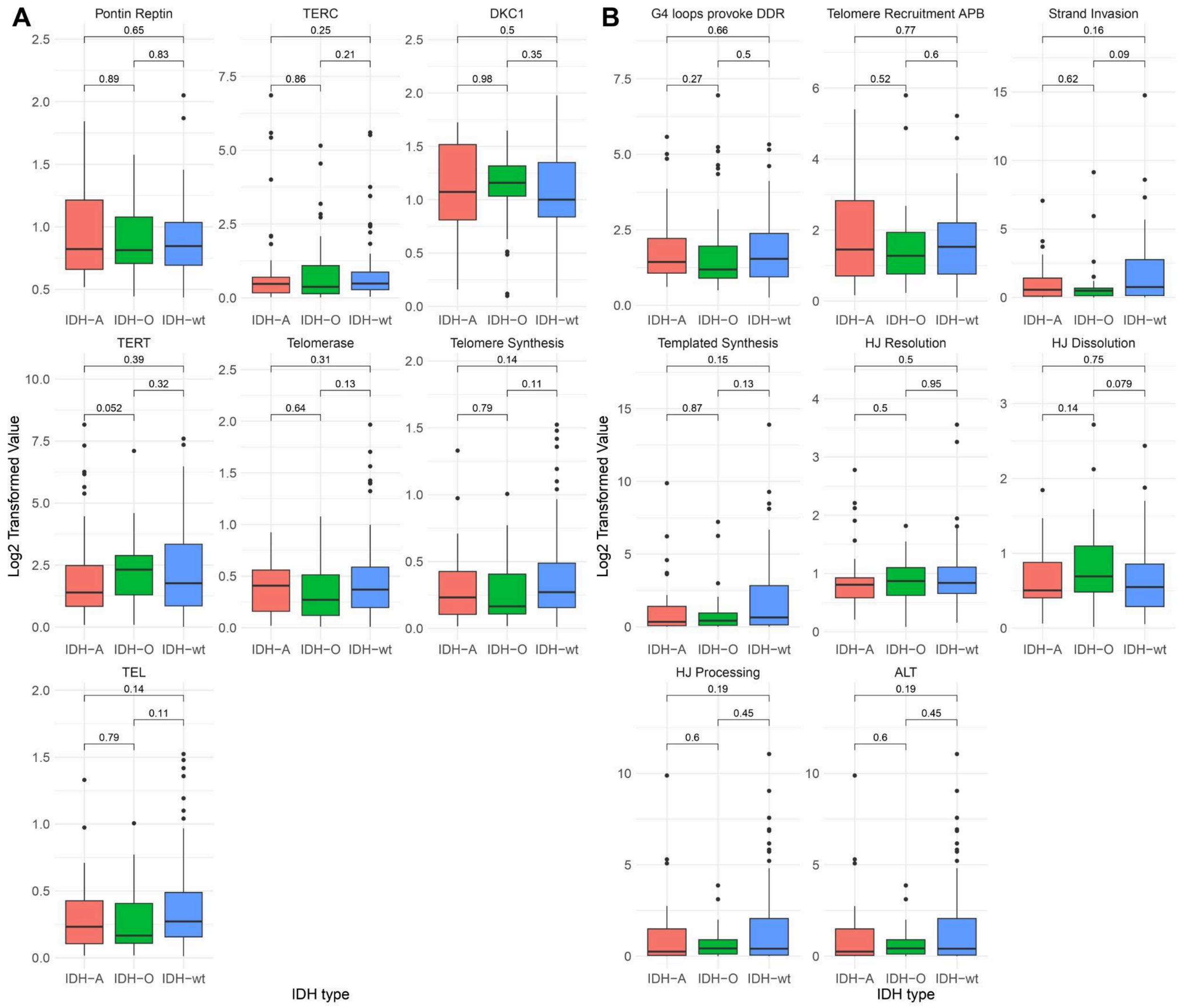

Figure. S5 The CGGA LGG TEL and ALT pathway branch activity across IDH subtypes and telomere length categories.

(A) The TEL and **(B)** The ALT pathway Log2-transformed PSF values of key branches across the IDH subtypes (IDH-A, IDH-O, IDH-wt). Data are stratified based on telomere length groups (Long TL in red and Short TL in blue). Statistical comparisons were performed between telomere length groups within each IDH subtype. Statistical significance was assessed using the Mann-Whitney U test.

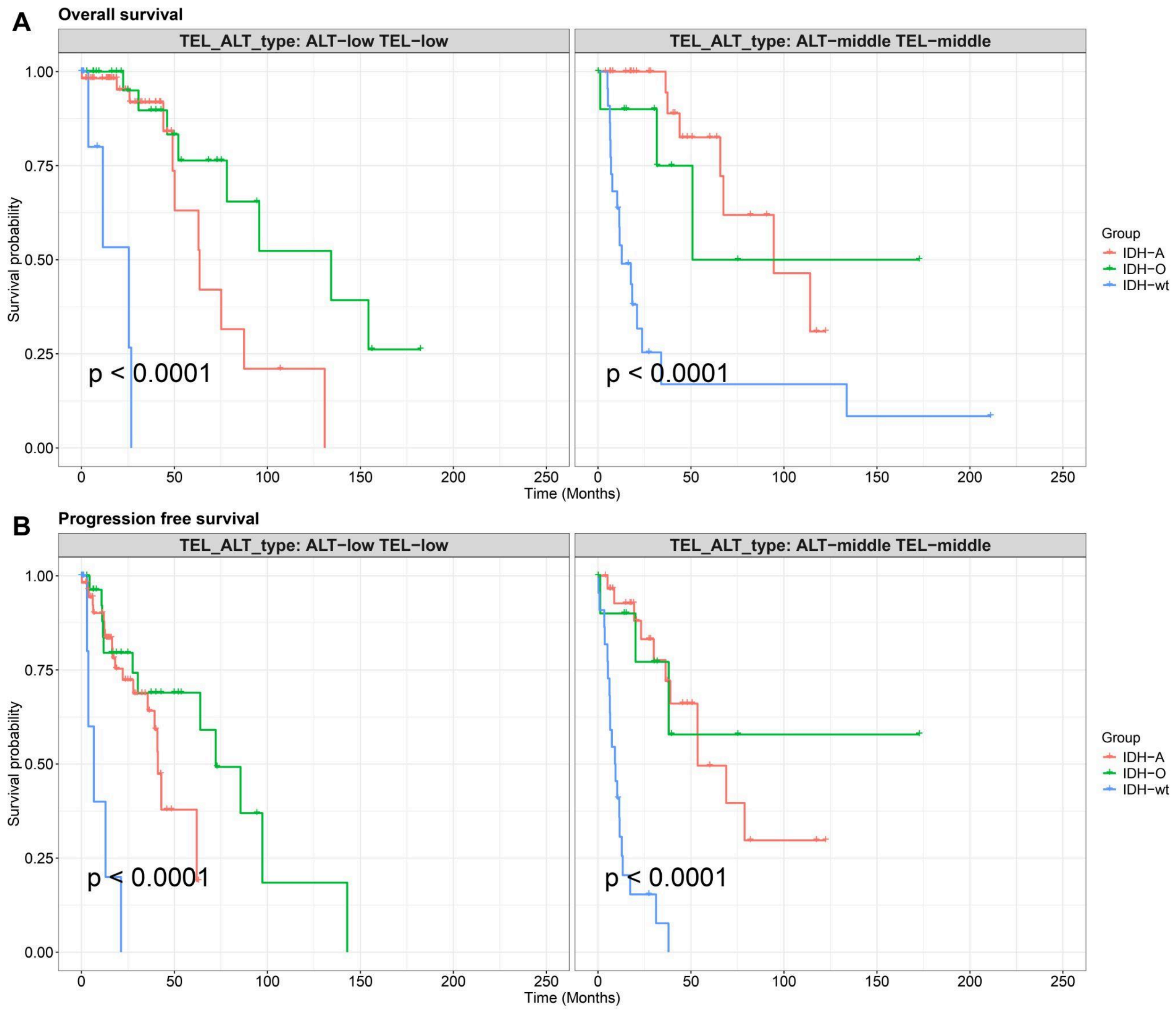

Figure. S6. Survival plots for IDH subtypes for TMM phenotype.

(A) Overall survival and (**B)** Progression-free survival curves. The pairwise log-rank test assessed the significance between TMM phenotype groups. Only TMM phenotypes with significant p-values are plotted.

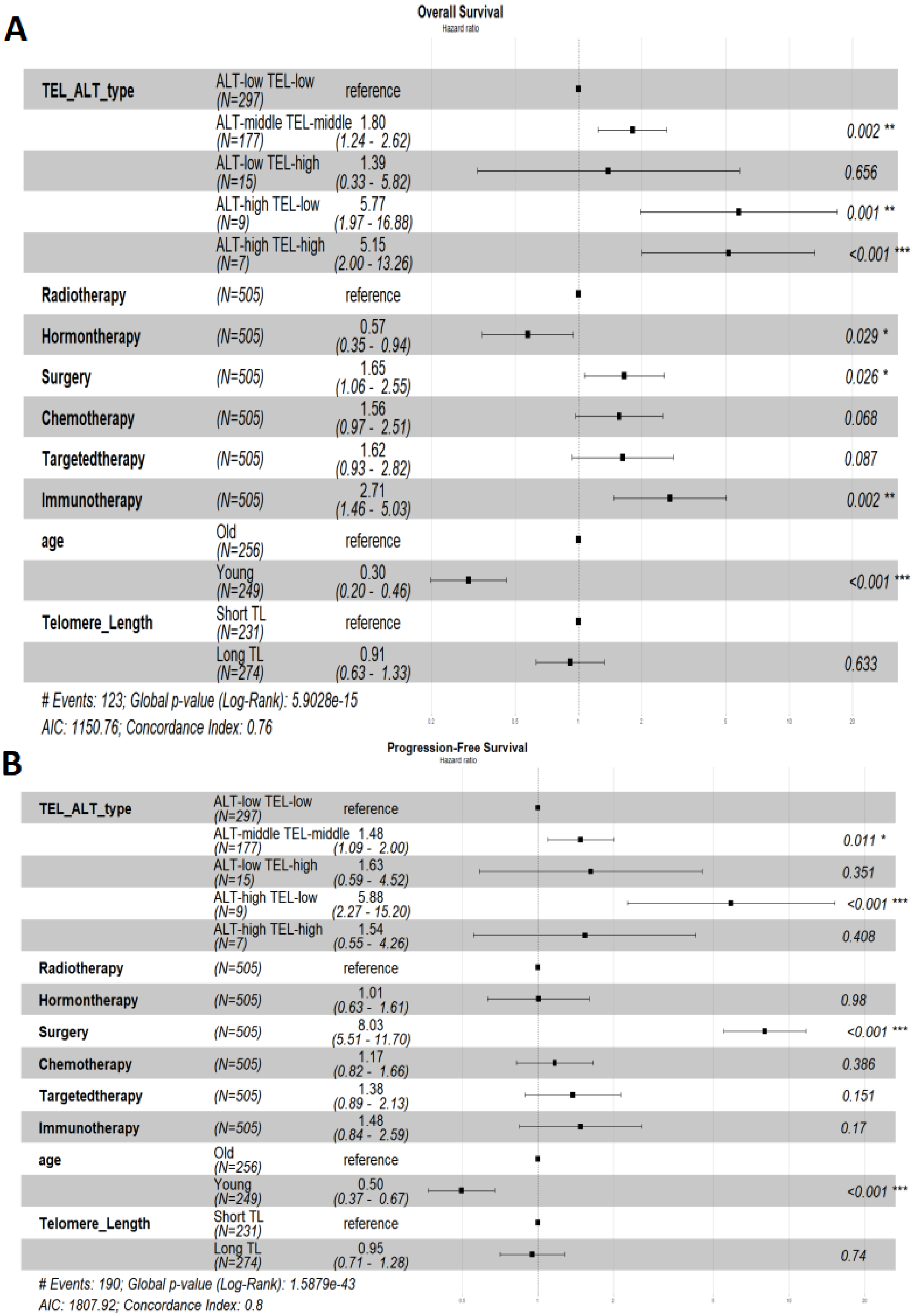
A
Overall Survival
Hazard ratio
TEL_ALT_type
ALT-low TEL-low (N=297) reference
ALT-middle TEL-middle (N=177) 1.80 (1.24 - 2.62) 0.002 **
ALT-low TEL-high (N=15) 1.39 (0.33 - 5.82) 0.656
ALT-high TEL-low (N=9) 5.77 (1.97 - 16.88) 0.001 **
ALT-high TEL-high (N=7) 5.15 (2.00 - 13.26) <0.001 ***
Radiotherapy (N=505) reference
Hormontherapy (N=505) 0.57 (0.35 - 0.94) 0.029 *
Surgery (N=505) 1.65 (1.06 - 2.55) 0.026 *
Chemotherapy (N=505) 1.56 (0.97 - 2.51) 0.068
Targetedtherapy (N=505) 1.62 (0.93 - 2.82) 0.087
Immunotherapy (N=505) 2.71 (1.46 - 5.03) 0.002 **
age
Old (N=256) reference
Young (N=249) 0.30 (0.20 - 0.46) <0.001 ***
Telomere_Length
Short TL (N=231) reference
Long TL (N=274) 0.91 (0.63 - 1.33) 0.633
Events: 123; Global p-value (Log-Rank): 5.9028e-15
AIC: 1150.76; Concordance Index: 0.76
B
Progression-Free Survival
Hazard ratio
TEL_ALT_type
ALT-low TEL-low (N=297) reference
ALT-middle TEL-middle (N=177) 1.48 (1.09 - 2.00) 0.011 *
ALT-low TEL-high (N=15) 1.63 (0.59 - 4.52) 0.351
ALT-high TEL-low (N=9) 5.88 (2.27 - 15.20) <0.001 ***
ALT-high TEL-high (N=7) 1.54 (0.55 - 4.26) 0.408
Radiotherapy (N=505) reference
Hormontherapy (N=505) 1.01 (0.63 - 1.61) 0.98
Surgery (N=505) 8.03 (5.51 - 11.70) <0.001 ***
Chemotherapy (N=505) 1.17 (0.82 - 1.66) 0.386
Targetedtherapy (N=505) 1.38 (0.89 - 2.13) 0.151
Immunotherapy (N=505) 1.48 (0.84 - 2.59) 0.17
age
Old (N=256) reference
Young (N=249) 0.50 (0.37 - 0.67) <0.001 ***
Telomere_Length
Short TL (N=231) reference
Long TL (N=274) 0.95 (0.71 - 1.28) 0.74
Events: 190; Global p-value (Log-Rank): 1.5879e-43
AIC: 1807.92; Concordance Index: 0.8

Figure. S7. Forest Plot of Hazard Ratios for Overall Survival (OS) and Progression-Free Survival (PFS) in relation to Clinical and Molecular Factors.

(A) The OS and (**B)** The PSF. The Cox proportional hazards model was applied to assess the impact of treatment types (Radiotherapy, Hormontherapy, Surgery, Chemotherapy, Targeted Therapy, and Immunotherapy), age, Telomere Length, and the TEL and ALT phenotype on survival outcomes.

ALT Pathway Activation Patterns in ATRX-Mutated Cancers: Insights Across Multiple Tumor Types

To investigate the role of ATRX mutations in ALT pathway activation, we analyzed ALT pathway activity across multiple cancer types. In the selected datasets, ATRX-mutated samples exhibited lower ALT PSF values in pancreatic adenocarcinoma (PAAD), kidney renal clear cell carcinoma (KIRC), and LGG, whereas in breast invasive carcinoma (BRCA), uterine corpus endometrial carcinoma (UCEC), and colon adenocarcinoma (COAD) displayed increased ALT pathway activity in ATRX-mutated samples. The BRCA, COAD, KIRC, PAAD, and UCEC IDH status were obtained from cBioportal (20). TMM-PSF data for these samples and ATRX mutation status information were derived from our previous studies (see main article references [13,39])
